# Identification of Potential New Genes Related to the SREBP Pathway in *Xanthophyllomyces dendrorhous*

**DOI:** 10.3390/biom14070778

**Published:** 2024-06-29

**Authors:** Maximiliano Venegas, Alejandro Durán, Sebastián Campusano, Salvador Barahona, Dionisia Sepúlveda, Marcelo Baeza, Víctor Cifuentes, Jennifer Alcaíno

**Affiliations:** 1 Departamento de Ciencias Ecológicas, Facultad de Ciencias, Universidad de Chile, Las Palmeras 3425, Santiago 7800003, Chile; maximiliano.venegas@ug.uchile.cl (M.V.);; 2 Facultad de Ciencias, Universidad de Chile, Las Palmeras 3425, Santiago 7800003, Chile

**Keywords:** SREBP/Sre1, random mutagenesis, mutant selection screening, carotenoids, mevalonate/MVA pathway

## Abstract

The sterol regulatory element-binding protein (SREBP) pathway is an integral cellular mechanism that regulates lipid homeostasis, in which transcriptional activator SREBPs regulate the expression of various genes. In the carotenogenic yeast *Xanthophyllomyces dendrorhous*, Sre1 (the yeast SREBP homolog) regulates lipid biosynthesis and carotenogenesis, among other processes. Despite the characterization of several components of the SREBP pathway across various eukaryotes, the specific elements of this pathway in *X. dendrorhous* remain largely unknown. This study aimed to explore the potential regulatory mechanisms of the SREBP pathway in *X. dendrorhous* using the strain CBS.*cyp61^-^* as a model, which is known to have Sre1 in its active state under standard culture conditions, resulting in a carotenoid-overproducing phenotype. This strain was subjected to random mutagenesis with N-methyl-N’-nitro-N-nitrosoguanidine (NTG), followed by a screening methodology that focused on identifying mutants with altered Sre1 activation phenotypes. Single-nucleotide polymorphism (SNP) analysis of 20 selected mutants detected 5439 single-nucleotide variants (SNVs), narrowing them down to 1327 SNPs of interest after a series of filters. Classification based on SNP impact identified 116 candidate genes, including 49 genes with high impact and 68 genes with deleterious moderate-impact mutations. BLAST, InterProScan, and gene ontology enrichment analyses highlighted 25 genes as potential participants in regulating Sre1 in *X. dendrorhous*. The key findings of this study include the identification of genes potentially encoding proteins involved in protein import/export to the nucleus, sterol biosynthesis, the ubiquitin–proteasome system, protein regulatory activities such as deacetylases, a subset of kinases and proteases, as well as transcription factors that could be influential in SREBP regulation. These findings are expected to significantly contribute to the current understanding of the intricate regulation of the transcription factor Sre1 in *X. dendrorhous*, providing valuable groundwork for future research and potential biotechnological applications.

## 1. Introduction

**Figure 1:**
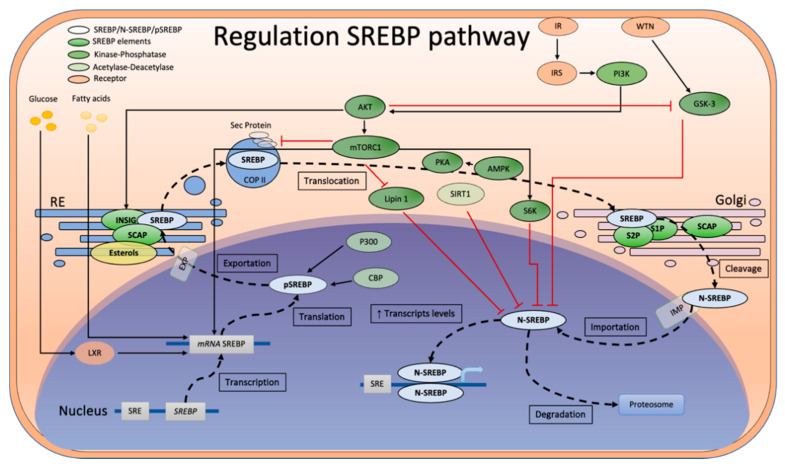
SREBP regulation mechanisms. A decline in sterol levels triggers the transport of the SREBP–Scap complex to the Golgi apparatus via COPII vesicles. At the Golgi apparatus, SREBP undergoes sequential cleavages by the proteases S1P and S2P, releasing the transcription factor N-SREBP. N-SREBP then translocates to the nucleus, where it binds to sterol regulatory elements (SREs) in the promoters of target genes. In addition, SREBP can be subjected to various post-translational modifications influencing its degradation or stabilization. Several kinases and phosphatases are known to regulate SREBP, including Phosphoinositide 3-kinase (PI3K), Glycogen synthase kinase 3 (GSK-3), protein kinase B (AKT), mammalian target of rapamycin complex 1 (mTORC1), protein kinase A (PKA), 5′AMP-activated protein kinase (AMPK), ribosomal S6 kinase (S6K), and lipin-1 phosphatase. Furthermore, acetylation by histone acetyltransferases p300 and CBP enhances SREBP stability as this modification competes with ubiquitination. Conversely, deacetylation by Sirtuin 1 (SIRT1) promotes SREBP degradation, thereby negatively regulating the expression of its target genes. (Figure adapted from [2,3]).

The regulation of mammalian lipid homeostasis depends on the transcription factor SREBP (sterol regulatory element-binding protein), a transcriptional activator. When sterol levels are sufficient, SREBP is anchored to the endoplasmic reticulum (ER) membrane via two transmembrane segments, where it interacts with the protein Scap (SREBP cleavage-activating protein) through its carboxyl-terminal domain (Figure 1). In turn, Scap, a sterol-binding protein, interacts with the protein INSIG (insulin-induced gene 1), retaining the SREBP–Scap complex at the ER membrane, thereby maintaining the SREBP transcription factor in its inactive state. However, when sterol levels in the membranes decrease, Scap undergoes a conformational change, disrupting its interaction with INSIG and enabling the transportation of the SREBP–Scap complex to the Golgi apparatus. Within the Golgi apparatus, SREBP undergoes sequential cleavage by the proteases S1P and S2P, releasing the amino-terminal domain (N-SREBP), representing the active form of the transcription factor. N-SREBP is then transported to the nucleus, where it binds to sterol regulatory elements (SREs) at the promoter region of its target genes, activating the transcription of genes involved in lipid synthesis and uptake, among other processes [1].

The yeast *Xanthophyllomyces dendrorhous* has emerged as a promising microbial platform for producing carotenoids and other isoprenoids, particularly astaxanthin, a xanthophyll with multiple biotechnological applications in the cosmetic, pharmaceutical, and aquaculture industries due to its potent antioxidant and pigmenting properties [4,5]. Several strategies have been employed with promising results to increase the yield of these valuable compounds, one of which is metabolic engineering [6,7,8]. This yeast has a functional SREBP pathway, which, when activated, increases the production of carotenoids [9]. To date, only two genes of the SREBP pathway have been described in *X. dendrorhous, SRE1* and *STP1*. The *SRE1* gene encodes the Sre1 protein, the yeast SREBP homolog, which exhibits the characteristic features of SREBP-type transcription factors. Furthermore, RNA-seq analyses and chromatin immunoprecipitation (ChIP) assays with the N-terminal domain of Sre1 (Sre1N) have indicated that Sre1 directly regulates genes involved in the mevalonate (MVA) pathway, as well as genes of ergosterol and astaxanthin biosynthesis, among others [10]. The *STP1* gene encodes the Stp1 protein, a homolog to the mammalian S2P protein that participates in the proteolytic activation of SREBPs [11]. Sre1 predominantly exists as an uncleaved, full-length protein in the wild-type strain under laboratory culture conditions. In contrast, under the same conditions, Sre1 is in its cleaved active form (Sre1N) [11] in the mutant strain CBS.*cyp61^-^*, which does not produce ergosterol and overproduces carotenoids [12]. Introducing a *sre1*^-^ mutation into strain CBS.*cyp61^-^* reverses the carotenoid overproduction phenotype, and replacing the *SRE1* gene in the wild-type strain with a truncated version of the gene encoding only Sre1N leads to increased production of carotenoids similar to those in strain CBS.*cyp61^-^* [9]. These findings support the SREBP pathway as a promising target for manipulation to enhance carotenoid and other isoprenoid production in *X. dendrorhous*.

Notably, mammalian INSIG and Scap homologs encoding genes have not yet been detected in the genome of *X. dendrorhous*. However, these observations are not unique to *X. dendrorhous*. For example, while the fission yeast *Schizosaccharomyces pombe* has an INSIG homolog, it has been demonstrated that it is not involved in the SREBP pathway in this yeast [13]. The presence of Scap is less prevalent in fungi and yeasts than is SREBP itself [14]. *S. pombe* has a Scap homolog that plays a role in the SREBP pathway [13], but *Aspergillus fumigatus* does not, despite having a functional SREBP homolog named SrbA [15,16]. In the basidiomycete *Cryptococcus neoformans*, both SREBP and Scap homologs have been identified and confirmed to play active roles in the SREBP pathway [17]. However, in *X. dendrorhous*, which is also a basidiomycete, a Scap homolog remains unknown, posing a challenge to understanding how SREBP homologs are activated without the sterol sensor Scap in organisms such as *X. dendrorhous*. In terms of SREBP homologs activating proteases, as in *X. dendrorhous*, *C. neoformans* has a functional S2P homolog involved in the activation of Sre1 [18], yet *A. fumigatus* and *S. pombe* lack this homolog. Instead, in these species, the activation of SREBP homologs requires a multi-protein complex named Dsc (defective for SREBP cleavage) and a rhomboid protease [19,20]. More recently, in the ascomycete fungus *Aspergillus nidulans,* a new component of the SREBP pathway was discovered; the protease SppA (signal peptide peptidase A), which is also involved in the intramembrane cleavage of SrbA [21]. These findings highlight the potential diversity in the components of the SREBP pathway across different fungal species. 

The regulation of SREBPs includes several mechanisms, including those summarized in Figure 1. Post-translational modifications, such as phosphorylation of SREBP by kinases AMPK (adenosine monophosphate-activated protein kinase) [22] and PKA (protein kinase A) [23], prevent its activation by retaining the transcription factor at the ER. Conversely, the master transcriptional regulator mTOR (mammalian target of rapamycin) phosphorylates several proteins, including the phosphatase lipin-1, which negatively regulates the active form of SREBP at the nuclear level [24], and kinases like S6K (S6 kinase) that negatively regulate SREBP when phosphorylated by mTOR [25]. In addition, other regulatory pathways, such as the Wnt signaling pathway involved in development and cellular homeostasis, indirectly regulate SREBP via GSK3 (glycogen synthase kinase 3), which is also involved in several signaling pathways, including those governing cell proliferation, glucose regulation, and apoptosis [26]. The phosphorylation of nuclear isoforms of SREBP by GSK3 promotes its ubiquitination, leading to SREBP degradation through the proteasome pathway [27]. In addition, the acetylation/deacetylation of SREBPs also influences their stability and interaction with coactivators and other regulatory proteins, subsequently affecting their processing, activation, and transcriptional function. Acetylation by p300 (E1A-binding protein p300) and CBP (CREB-binding protein), both of which exhibit histone acetyltransferase (HAT) activity, has been shown to influence SREBP stability [28]. On the other hand, Sirtuin 1 deacetylase SIRT1 targets lysine residues when acetylated, thereby increasing SREBP susceptibility to ubiquitination and subsequent degradation [29]. In addition, ubiquitin ligases like GP78 and TRC8 polyubiquitinate SREBPs, leading to their degradation via the proteasome. Moreover, fatty acids and glucose can also regulate SREBP activity. These metabolites function through the LXR receptor pathway, where LXRs act as nuclear sensors of lipids. Upon activation, LXRs form a complex that induces the transcription of numerous target genes, including SREBPs [30,31].

This study investigated potential regulatory mechanisms of the SREBP pathway in fungi, particularly in *X. dendrorhous.* Using random mutagenesis on the strain CBS.*cyp61^-^*, which overproduces carotenoids due to the transcription factor Sre1 being in its active state, we selected mutants displaying a *sre1*- phenotype. These mutants exhibited wild-type pigmentation and sensitivity to clotrimazole. Single-nucleotide polymorphism (SNP) analysis was conducted on these mutants to identify genes potentially responsible for the selected phenotype, which may play a role in the SREBP pathway in *X. dendrorhous*.

## 2. Materials and Methods

### 2.1. Strains, Plasmids, Primers, and Enzymes

The plasmids and strains used in this study are listed in Table 1. For molecular cloning and plasmid propagation, the *E. coli* DH5α strain [32] was used and cultivated in LB medium (1% tryptone, 0.5% yeast extract, 0.5% NaCl) with constant agitation at 37 °C. To select transformants, semi-solid LB medium (1.5% agar) supplemented with ampicillin (100 µg/mL) and X-gal (120 µg/mL) was used. *X. dendrorhous* strains were cultivated in YM medium (0.3% yeast extract, 0.3% malt extract, 0.5% bactopeptone) supplemented with 1% glucose under constant agitation at 22 °C. Yeast transformants were selected using a semi-solid YM medium (1.5% agar) supplemented with zeocin (0.75 µg/mL). The primers for PCR reactions and sequencing are listed in Supplementary Table S1 and were synthesized by Integrated DNA Technologies (Coralville, IA, USA). The enzymes used in this study included T4 DNA ligase, restriction endonucleases, Maxima reverse transcriptase, PfuUltraII Fusion HS DNA polymerase, DNase I, RNase A, T4 polynucleotide kinase, and FastAP thermosensitive alkaline phosphatase, obtained from Agilent Technologies (Santa Clara, CA, USA), Thermo Fisher Scientific (Waltham, MA, USA), and Life Technologies (Carlsbad, CA, USA), and used according to the manufacturer’s instructions.

### 2.2. X. dendrorhous Transformation

The *STP1* and *SRE1* genes were replaced with the wild-type versions of the gene fused to a cassette that confers resistance to zeocin for transformant selection using the plasmids pBS-*gSTP1*^up-down^ and pXd-*gSRE1*-zeo, respectively (Table 1). *X. dendrorhous* was transformed by electroporation using a GenePulser XcellTM (BioRad, Hercules, CA, USA). Electrocompetent cells were prepared from cultures in YM medium at exponential growth phase with an OD_600_ of 4–5 [33]. Electroporation was performed using 6 μL of transforming DNA (10 μg) employing a 2 mm cuvette and the following conditions: 450 V, 125 μF, and 600 Ω. After the pulse, 1 mL of YM was added to the cells, which were incubated for 4 h at 22 °C before seeding on YM-agar 1.5% plates with the respective antibiotic to select the transformants.

### 2.3. Random Mutagenesis

Random mutagenesis was performed with N-methyl-N’-nitro-N-nitrosoguanidine (NTG) as previously described [34,35,36]. For standardization, the CBS.*cyp61^-^ X. dendrohous* strain was cultured in 500 mL flasks containing 100 mL of YM medium (1:5 ratio), which were inoculated at a 1:100 ratio from a 48 h yeast culture. The cultures were grown to reach an OD_600_ of between 0.4 and 0.6. The cells were collected and resuspended in 20 mL of medium, and NTG was added to reach a final concentration of 100 μg/mL, 500 μg/mL, or 1000 μg/mL, and incubated for 30 min at room temperature. Following treatment, the cells were collected by centrifugation at 14,000 rpm for 5 min, washed twice with water, and suspended in 5 mL of water. Serial dilutions were plated on semi-solid YM medium and incubated at 22 °C for 3 to 4 days until colony development. The survival percentage at different NTG concentrations was estimated by calculating the colony forming units (CFUs) per mL and comparing this to the control with no NTG exposure. The random mutagenesis experiment was performed on strain CBS.*cyp61^-^.FLAG.SRE1* exposed for 30 min to the selected NTG concentration (100 μg/mL). Colonies exhibiting wild-type pigmentation were selected and seeded on YM and YM–clotrimazole (0.15 μg/mL) plates. Colonies that maintained wild-type pigmentation and exhibited sensitivity to clotrimazole were selected for subsequent experiments.

### 2.4. Purification and Extraction of Nucleic Acids

Plasmid DNA was purified using the GeneJET Plasmid Miniprep Kit from Thermo Fisher Scientific (Waltham, MA, USA), following the manufacturer’s instructions. DNA purification from agarose gels was performed using the silica bead method [37], and yield was evaluated in 1% agarose gels. *X. dendrorhous* genomic DNA extraction was conducted using glass beads [32] for routine applications. However, for genome sequencing using Illumina HiSeq X Ten, 2 × 150 bp, high–molecular-weight and -integrity DNA was obtained. For this, DNA was extracted from 3-day yeast cultures grown in YM medium at 22 °C. The cultures were fractionated into 2 mL cryotubes and lyophilized at −84 °C for 24 h using a Labconco Freezone 2.5L lyophilizer. The lyophilized samples were then mechanically disrupted for 5 min using a MiniBeadBeater-16 without adding buffer, after which 600 μL of lysis buffer from the Tissue DNA Extraction Kit (Abbott mSample Preparation System 04J70-24, Promega Corporation, Madison, WI, USA) was added. The samples were vortexed for 5 s and then centrifuged for 10 min at 14,000 rpm. Supernatants were collected, and 30 μL of magnetic beads (Promega) were added. Samples were mixed for 10 min with constant rotation at 10 rpm using an ELMI Intelli-Mixer RM-2L rotator equipment and then placed on a magnetic rack to remove the supernatant without discarding the magnetic beads. The beads were then washed once with 400 μL of isopropanol and twice with 1 mL of 75% ethanol, air-dried at room temperature for 10 min, and the DNA was eluted with 50–100 μL of 1:10 TE (1 mM Tris, 10 mM EDTA). DNA concentration and quality were determined by fluorometry using the Qubit 4 from Thermo Fisher Scientific (Waltham, MA, USA) with the Qubit dsDNA HS kit. The DNA was precipitated using ethanol and sodium acetate upon achieving the desired concentrations.

For RNA extraction from yeast, 5 mL of culture was used. The cell pellet was suspended in 200 μL of lysis buffer (0.5 M sodium acetate pH 5.5, 10% SDS, 0.5 M EDTA pH 8.0), and 100 μL of 0.5 mm diameter glass microbeads were added. The mixture was placed in a BioSpec Mini-Beadbeater-16 cell disruptor for 3 min, and 800 μL of TriReagent solution from Life Technologies (Carlsbad, CA, USA) was added. Next, the mixture was vortexed for 7 min and homogenized at room temperature for 10 min at 8 rpm in an Intelli Mixer RM-2L large (ELMI). Subsequently, 200 μL of chloroform was added and homogenized again for 10 min. The solution was then centrifuged at 14,000 rpm for 10 min at 4 °C, and the aqueous phase was collected. To this, 250 μL of water and 550 μL of isopropanol were added, homogenized for another 10 min, and then centrifuged at 14,000 rpm for 15 min at 4 °C. The resulting pellet was washed with 1 mL of 70% ethanol and centrifuged for 6 min at 14,000 rpm at 4 °C. The supernatant was discarded, and the pellet was air-dried and finally suspended in 10 μL of nuclease-free water.

### 2.5. DNA Amplification, cDNA Synthesis (RT), and Real-Time PCR (qPCR)

The PCR reactions included 1X PCR buffer (500 mM KCl, 200 mM Tris-HCl pH 8.4), 2 mM MgCl_2_, 0.2 µM of each dNTP (dATP, dTTP, dGTP, dCTP), 1 µM of each primer, 1U of DNA polymerase enzyme, and between 10 and 20 ng of template DNA. DNA amplification was performed using an Applied Biosystem (Waltham, MA, USA) 2720 thermocycler initiating with denaturation at 94 °C for 3 min, followed by 30 cycles of denaturation at 94 °C for 30 s, annealing at 55 °C for 30 s, and extension at 72 °C with time adjusted to the size of the amplicon. A final extension step at 72 °C for 10 min concluded the reaction, and the samples were kept at 4 °C until use.

The RNA was treated with DNase I for cDNA synthesis according to the enzyme supplier’s instructions. Reverse transcription (RT) was performed using the Maxima Reverse Transcriptase enzyme from Thermo Fisher Scientific (Waltham, MA, USA) in a final volume of 20 µL, using 5 µg of RNA.

According to the manufacturer’s instructions, qPCR reactions were performed using the SsoAdvanced Universal SYBR Green Supermix Kit from BioRad (Hercules, CA, USA). Each reaction included 10 µL of the kit mix, 8 µL of sterile water, 1 µL of cDNA, and 1 µL of 10 µM primer mix, and was amplified in a BIO RAD C1000 touch thermal cycler CFX96 Real-Time System. Transcript levels were quantified by normalizing Ct (threshold cycle) values to the housekeeping ACT gene (Genbank: X89898.1) of *X. dendrorhous* and expressed relative to control conditions using the 2^-∆∆*C*t^ method [38].

For the sequencing of the *SRE1* and *STP1* genes, primers were designed to allow the amplification of the entire gene (Supplementary Table S1). Amplicons were sequenced at Macrogen (Seoul, Republic of Korea), and the reads were analyzed with Geneious v8.0.0.

### 2.6. Bioinformatic Analyses, Genome Mapping, SNP Calling, and Annotation

General bioinformatic analysis such as primer design, gene and protein analysis, and imaging were performed using Geneious v8.0.0. Protein bioinformatic characterizations were conducted with InterProScan [39] to search conserved domains and protein features.

Genome sequencing of selected mutants was performed using the services from Omics2view.consulting (https://www.omics2view.consulting/, accessed on 25 June 2024) with Illumina HiSeq X Ten, 2 × 150 bp (accession number: PRJNA1098865). The quality of the obtained raw *reads* was verified using FastQC (q > 30) [40]. Subsequently, low-quality *reads* and adapters were removed using Cutadapt [41], and after *trimming*, another quality analysis with FastQC was performed to reevaluate read quality prior to mapping the filtered reads onto the PacBio+Illumina available genome of *X. dendrorhous* (accession number: GCA_014706385.1). The genome annotation [42], based on BLASTp analysis against the NCBI reference sequence database (Protein RefSeq), was conducted with Augustus [43] and BRAKER [2] and included as supplemental information (Supplementary Table S8). For the latter, the transcriptomic data from GEO Series (accession number: GSE152739) from the wild-type strain CBS 6938 cultured under standard conditions were used, and the genome was indexed using the Java packages from GATK [44]. Once the genome was indexed, the filtered reads were mapped using the Burrow-Wheeler Aligner (BWA) [45], resulting in a sequence alignment/map (SAM) file. This SAM file was transformed into a binary alignment map (BAM) using SAMtools from the different mappings to reduce the file size. Upon obtaining the initial mapping draft, potential duplicate reads usually generated during read-to-genome mappings were removed using MarkDuplicatesSpark from BAM files [44].

Once the corrected BAM mapping file was obtained, the SNP calling process (SNP call) was performed using HaplotypeCaller-GVCF from the GATK package. This tool identifies single-nucleotide variants (SNVs) in reads mapped to different genome coordinates, resulting in a GVCF (genomic variant call format) file containing raw information about the identified SNV. Once this file was obtained, the SNP annotation was performed using SnpEff [46], which enables the conversion of the raw and coordinate information into data tables containing specific SNV positions, annotations of SNV effects at the protein level, impact type annotations (Supplementary Table S2), altered gene codes, and affected specific amino acids, among others. Subsequently, these tables were transformed into the TSV format and manipulated in Excel to filter out only those SNVs identified in coding regions (CDS) to facilitate data analysis. Finally, the Sorting Intolerant From Tolerant (SIFT) [47] tool was used to analyze moderate-impact mutations.

Protein sequences with high- and moderate-impact SNPs were analyzed using BLAST2GO to assign GO terms to each protein based on the similarities found and the information in the Gene Ontology database. The results were visualized for each category: molecular function, cellular component, and biological process using Google Sheets.

The RNA-seq analyses were conducted using reads from strains CBS 6938 and CBS.*FLAG.SRE1N* obtained from SRA Bioproject PRJNA517352 [10]. For this, the reads were trimmed using Cutadapt and verified through FastQC, like the genomic reads (q > 30), and mapped to the reference genome (accession number: GCA_014706385.1) with Bowtie2 2.5.3 [48]. Properly aligned paired reads were filtered with SAMtools 1.20 [49]. The R package Rsubread 2.16.1 [50] was used for reads counting overlapping each annotated gene, and differential expression analysis was carried out with DESeq2 1.42.1 [51] in R 4.3.3.

## 3. Results

### 3.1. Evaluation of Mutagenesis Conditions and Effectiveness of Screening for SREBP Pathway Mutants

The *X. dendrorhous* strain CBS.*cyp61*^-^ overproduces carotenoids, and this phenotype is attributable to the activation of Sre1, as *sre1*^-^ [9] and *stp1*^-^ [11] mutants derived from this strain exhibit a wild-type color phenotype. Therefore, mutants derived from strain CBS.*cyp61*^-^ with a wild-type color phenotype are potential candidates for mutants of the SREBP pathway. Additionally, while strain CBS.*cyp61*^-^ shows resistance to azole compounds, mutants of the SREBP pathway, such as *sre1*^-^ and *stp1*^-^, do not, as the SREBP pathway mediates resistance to these compounds in fungi [13,52,53]. Given these traits, strain CBS.*cyp61*^-^ serves as a valuable model for identifying genes involved in the SREBP pathway through random mutagenesis experiments, with mutant selection facilitated by rapid visual screening for pigmentation changes and azole sensitivity on agar plates.

Random mutagenesis was performed using NTG, a mutagenic agent known to primarily induce transition-type nucleotide changes and, to a lesser extent, deletions [54,55]. The strain was exposed to the mutagen for 30 min at final concentrations of 0, 100, 500, and 1000 µg/mL, obtaining survival percentages of 41.0%, 5.4%, and 0.7%, respectively. Colonies reverting to wild-type pigmentation were observed after treatment with NTG at 100 µg/mL, the concentration selected for subsequent mutagenesis experiments.

**Figure 2:**
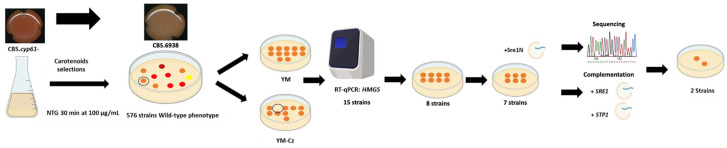
*X. dendrorhous* SREBP pathway mutant selection workflow and results. The CBS.*cyp61^-^* strain was exposed to 100 μg/mL of NTG for 30 min. Five hundred seventy-six colonies that displayed wild-type pigmentation were selected and seeded in replicas in both YM and YM + 0.15 μg/mL of clotrimazole (YM-Cz) plates. Fifteen colonies sensible to clotrimazole were recovered and the relative expression of the *HMGS* gene was analyzed by RT-qPCR. Eight strains had comparable *HMGS* transcript levels to the wild-type strain, which were transformed with the Sre1N gene version. Seven strains recovered the CBS.*cyp61^-^* phenotype, which were then transformed with the native versions of the *SRE1* and *STP1* genes, according to the mutated gene they had. The *SRE1* native gene complemented the strain having mutations in the *SRE1* gene, and the *STP1* native gene complemented the four strains having mutations in *STP1*. The screening and analysis confirmed that the selection method allowed the isolation of mutants of the SREBP pathway, and two mutants are potential mutants of unknown genes from the SREBP pathway in *X. dendrohous*.

**Figure 3:**
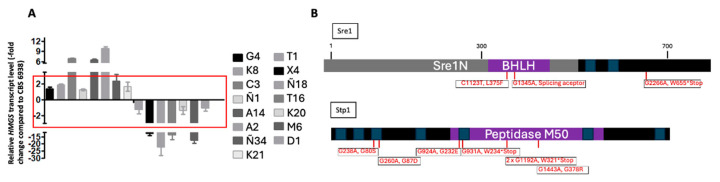
Analysis of selected mutants. (**A**) Relative expression of the *HMGS* [GenBank: MK368600] gene evaluated by RT-qPCR after 120 h of culture in YM medium with constant agitation and normalized to the housekeeping β-actin gene [GenBank: X89898.1]. Values are the mean ± standard deviation of three technical replicates. Relative expression fold changes between 2 and −2 are outlined with a red box. (**B**) Identified mutations and consequences in the Sre1 and Stp1 proteins (red). Transmembrane segments were predicted with THMMM v2.0 (blue), and intrinsic conserved domains were predicted with InterProScan analysis (purple): bHLH (PF00010) in Sre1 and peptidase M50 (IPR008915) in Stp1. The Sre1N domain is represented in gray.

A total of 576 colonies that exhibited wild-type pigmentation were initially selected (Figure 2). Of these colonies, 15 were further chosen as they were also sensitive to 0.15 µg/mL of clotrimazole. To determine if these 15 colonies could be potential mutants of the SREBP pathway, the transcript level of the 3-hydroxy-3-methylglutaryl-CoA synthase gene (*HMGS*) of the mevalonate pathway was evaluated using RT-qPCR. The *HMGS* gene is a known target of Sre1 in *X. dendrorhous,* and it shows a pronounced change in transcript levels between strains with the active Sre1N transcription factor and with a *sre1*^-^ mutation [9,10]. Consequently, the *HMGS* gene transcript level serves as an indicator of Sre1 activation in *X. dendrorhous*. Among the 15 evaluated strains, eight displayed *HMGS* transcript levels comparable to those in the wild-type strain (with a fold change between 2 and −2) (Figure 3A). This result suggests that Sre1 is possibly not activated in these strains under the given culture conditions compared to strain CBS.*cyp61*^-^, indicating that they are potential SREBP pathway mutants. To evaluate this possibility, the native *SRE1* gene in the eight selected mutants was replaced with a version expressing the active form of Sre1 (Sre1N), which does not require proteolytic activation. In seven strains, introducing Sre1N reversed the wild-type pigmentation and clotrimazole sensitivity to the overproduction of carotenoids and resistance phenotype as seen in the original CBS.*cyp61*^-^ strain. This last result suggests that these mutants likely have defects in activating Sre1 or in the Sre1 protein itself and were considered for subsequent analyses. Subsequently, the *SRE1* and *STP1* genes in the seven selected mutants were amplified by PCR and sequenced. Five strains harbored mutations in these genes; one was a *sre1*^-^ mutant, and four were *stp1*^-^ mutants (Table 2), confirming the efficacy of the selection strategy as *sre1*^-^ and *stp1*^-^ mutants were expected as controls of the screening method for selecting *X. dendrorhous* SREBP pathway mutants. However, as not all the mutations detected in the *SRE1* and *STP1* genes may necessarily impair the protein function, the seven mutants were transformed with the vectors pXd-g*SRE1*-zeo or pBS-g*STP1*^up-down^ to replace the mutated *SRE1* or *STP1* genes, respectively, with the wild-type versions of the genes. The transformation with the native genes restored the carotenoid overproduction and clotrimazole resistance phenotypes of the original CBS.*cyp61*^-^ strain in the corresponding gene mutants, corroborating the sequencing results. Table 2 and Figure 2 summarize the results obtained during the assays conducted to evaluate the effectiveness of mutant screening and selection.

### 3.2. CBS.cyp61^-^.SRE1.FLAG Strain Mutation and SREBP Pathway Mutant Selection

After confirming the effectiveness of the mutagenesis and the SREBP pathway mutant selection strategy, a new round of mutagenesis was conducted using strain CBS.*cyp61*^-^*.SRE1.FLAG*. This strain exhibits the same phenotype regarding pigmentation and resistance to clotrimazole as strain CBS.*cyp61*^-^ and shares a similar genetic background. However, they differ in that strain CBS.*cyp61*^-^*.SRE1.FLAG* expresses the Sre1 protein fused to a 3xFlag epitope at its N-terminal end [11]. This modification could facilitate further assays on Sre1 in the resulting mutants in future studies. After mutagenesis, 1,500 colonies displaying wild-type pigmentation were isolated; among them, 28 were sensitive to clotrimazole. Both the *SRE1* and *STP1* genes from these colonies were amplified by PCR and sequenced. Mutations in the *STP1* gene were found in six mutants (one mutant harbored two mutations in this gene) and in the *SRE1* gene in three mutants (Figure 3B). The ten identified mutations in these genes included nine G/A transitions (two mutants shared the same G/A transition) and one C/T transition. The detected mutations included a splicing acceptor site mutation (in *SRE1*), four resulted in nonsense mutations (in both *SRE1* and *STP1*), and five missense mutations (in both *SRE1* and *STP1*) affecting key motifs of the encoded proteins (Figure 3B). Nineteen of the selected mutants did not harbor mutations in *STP1* or *SRE1*, suggesting that they could be mutants of other genes associated with the SREBP pathway in *X. dendrorhous*. Genomic DNA from these 19 mutants, along with one of the mutants derived from the CBS.*cyp61-* strain mutagenesis (mutant K20), was extracted and sequenced using Illumina technology to perform SNP (single-nucleotide polymorphism) analysis.

The SNP analysis was conducted following the Broad Institute Genome Analysis Tool Kit (GATK) guidelines, as mentioned in the materials and methods section. A total of 5439 single-nucleotide variants (SNVs) were identified across the 20 strains (Supplementary Table S3), with 49.5% of all SNVs located in coding sequence (CDS) regions. Among the mutations in CDS, 83.2% were SNPs, and 81.3% of these were transitions. Within the transitions, the most prevalent were G/A and C/T transitions, corresponding to 37.4% and 39.9% of all SNPs, respectively.

Limited genomic data are available for *X. dendrorhous*; only two wild-type strain CBS 6938 genomes are available. For this reason, while acknowledging the risk of losing pertinent information, several filters were applied to simplify the SNV analysis. First, as our focus was to identify genes encoding proteins related to the SREBP pathway, SNVs in intergenic regions were excluded despite their potential to affect regulatory sequences that could contribute to the selected phenotype. This step reduced the SNV count to 3011 for analysis. Second, deletions and insertions impacting repeated regions and observed across multiple strains were omitted to avoid possible artifacts from mutant sequencing or the reference genome. This filtration left 1838 SNPs. Third, due to the low likelihood of identical mutations in different strains arising from random mutagenesis, any identical SNP identified at the same position in more than five strains was not considered, leaving 1327 SNPs (Supplementary Table S4). These 1327 SNPs were then classified according to the mutation impact using SnpEff as mentioned in materials and methods, identifying 53 SNPs of high impact (in 49 genes including 41.2% stop gained, 5.9% start lost, 15.7% splice acceptor variants and intron variants, 37.3% splice donor variants and intron variants), 611 of moderate impact (in 505 genes including 97.1% missense variants, 2.9% missense variants and splice region variants), 327 of modifier impact (in 261 genes including 100% intron variants), and 338 of low impact (257 genes including 89.3% synonymous variants, 8.3% splice region variants and intron variants, 1.7% splice region variants and synonymous variants). Moderate-impact SNPs were mainly responsible for amino acid changes at specific positions, which may or may not affect the protein function. Therefore, the SIFT tool was employed on the group of genes with moderate-impact SNPs to determine SNPs with a potentially deleterious effect (SIFT score < 0.05). For further analysis, genes having high (49 genes, Table 3 and Supplementary Table S5) or deleterious moderate (68 genes, Table 4 and Supplementary Table S6) impact mutations were considered potential contributors to the selected phenotype of the mutant strains. Then, BLAST, InterProScan, and enrichment analyses based on Gene Ontology were performed on these genes, classifying them by cellular component (C terms), biological process (P terms), and molecular function (F terms).

In genes with high-impact mutations, the term “integral component of membrane” (GO:0016021) was highlighted as the most prevalent, constituting 42.86% of the identified cellular component terms, followed by “nuclear periphery” (GO:0034399) representing 9.52% of the terms (Supplementary Figure S1). Among the molecular functions, “zinc ion binding” (GO:0008270) and “protein binding” (GO:0005515) were the most abundant categories, each accounting for 7.55% of the total, followed by “ATP binding” (GO:0005524) representing 5.66% of the identified F terms. The functions “DNA binding” (GO:0003677), “DNA-binding transcription factor activity, RNA polymerase II-specific” (GO:0000981), “metalloendopeptidase activity” (GO:0004222), “nucleic acid binding” (GO:0003676), and “oxidoreductase activity” (GO:0016491) each compromised 3.77% of the F terms (Supplementary Figure S2). Finally, in terms of biological processes, “proteolysis” (GO:0006508) and “oxidation-reduction process” (GO:0098869) were the most represented, both accounting for 6.82% of the total identified P terms, followed by “regulation of transcription by RNA polymerase II” (GO:0006357) that constituted 4.55% of the P terms (Supplementary Figure S3).

Among the terms related to cellular components on genes with potentially deleterious moderate-impact mutations, the most prominent ones in decreasing order were “cytosol” (GO:0005829, 6.3%), “integral component of membrane” (GO:0016021, 6.3%), “cytoplasm” (GO:0005737, 3.8%), “microtubule” (GO:0005874, 3.8%), “mitochondrial outer membrane” (GO:0005741, 3.7%), and “spindle pole” (GO:0000922, 3.8%) (Supplementary Figure S4). Regarding molecular functions, the most abundant ones listed in descending order, were “ATP binding” (GO:0005524, 6.6%), “GTP binding” (GO:0005525, 3.6%), “protein binding” (GO:0005515, 3.6%), “metal ion binding” (GO:0046872, 3.6%) “GTPase activity” (GO:0003924, 2.9%), “nucleic acid binding” (GO:0003676, 2.9%), “magnesium ion binding” (GO:0000287, 2.2%), “microtubule binding” (GO:0008017, 2.2%), “kinase activity” (GO:0016301, 2.2%) and “zinc ion binding” (GO:0008270, 2.2%) (Supplementary Figure S5). Finally, concerning biological processes, the most abundant were “oxidation-reduction process” (GO:0055114, 4.1%), “protein peptidyl-prolyl isomerization” (GO:0000413, 2.5%), “ergosterol biosynthetic process” (GO:0006696, 2.5%), and “protein homooligomerization” (GO:0051260, 2.4%) (Supplementary Figure S6).

Finally, considering the potential autoregulatory role of Sre1, where Sre1N might activate the transcription of genes encoding its regulators, a transcriptomic analysis focusing on genes with high- and potentially deleterious moderate-impact mutations was performed. For this, previously obtained RNA-seq data [10] were analyzed from a strain expressing the activated transcription factor Sre1N (CBS*.FLAG*.*SRE1N*) and the wild-type strain CBS 6938. Among the genes with high-impact mutations, five genes were overexpressed in strain CBS*.FLAG.SRE1N* compared to the wild-type strain (log2 > 1, Table 3) and 11 genes with deleterious moderate-impact mutations showed higher expression in the same analysis (log2 > 1, Table 4).

**Figure 4:**
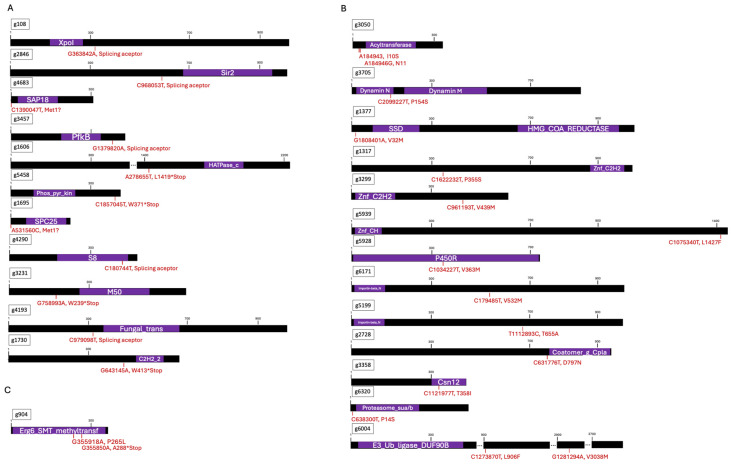
Analysis of the 25 genes selected as potential candidates for Sre1 Regulation. Genes with (**A**) high-impact, (**B**) deleterious moderate-impact, and (**C**) high- and deleterious moderate-impact mutations. Mutation site (red) and conserved domains predicted with InterProScan analysis (purple): XpoI (Exportin-1/Importin-b-like) (IPR013598), Sir2 (IPR026587), PfkB (IPR011611), HATPase_c (IPR003594), Phos_pyr_kin (IPR013749), SPC25 (IPR009582), Fungal_trans (IPR007219), C2H2 (IPR013087), SAP18 (IPR010516), M50 (IPR008915), Acyltransferase (IPR004552), Dynamin N (IPR045063), Dynamin M (IPR000375), HMG_COA_REDUCTASE (IPR002202), SSD (IPR000731), Znf_C2H2 (IPR013087), Znf_CH (IPR011016), P450R (IPR023208), Importin-beta_N (IPR001494), Coatomer_g_Cpla (IPR032154), Csn12 (IPR045114), Proteasome_sua/b (IPR001353), E3_Ub_ligase_DUF908 (IPR010309), and Erg6_SMT_methyltransf (IPR050447). In cases where two alternative transcripts were predicted (g2846 and g4193), only protein deduced from transcript 1 (.t1) was considered for mapping the mutations.

In summary, our analysis identified 49 genes with high-impact mutations (Table 3) and, according to the SIFT analysis, 68 genes with moderate-impact mutations (Table 4) that could have deleterious outcomes. From the results from the BLAST, InterproScan, and Gene Ontology analyses, 25 genes (Figure 4) emerged as potential candidates for regulating the SREBP pathway in *X. dendrorhous*. Of particular interest is gene g904, as two of the selected mutants had mutations in this gene: one of them (Sample_003) had a high-impact mutation (Arg288*), and the other (Sample_006), a deleterious moderate-impact mutation (Pro265Leu) and a modifier mutation (Intron variant). The gene is associated with various GO terms, including lipid droplet localization (C: term), sterol C-24-methyltransferase activity (F: term), and identical protein binding (F: term), as well as roles in ergosterol biosynthetic (P: term) and methylation (P: term). In addition, bioinformatic predictions strongly suggest that g904 encodes the Erg6 (sterol 24-C-methyltransferase) protein, known for its role in the biosynthesis of ergosterol, specifically in transforming zymosterol into fecosterol. Notably, this gene is overexpressed in the strain possessing the active Sre1N transcription factor compared to the wild-type. These findings collectively suggest that mutations in g904 could be responsible for the phenotype of some of the selected mutants in this work, highlighting its potential to regulate Sre1 directly or indirectly in *X. dendrorhous* and underscoring its significance in the regulatory network governing lipid metabolism in this organism.

## 4. Discussion

The current study aimed to explore potential regulatory mechanisms of the SREBP pathway in the carotenogenic yeast *X. dendrorhous* and to contribute to a deeper understanding of the intricate regulation of the transcription factor Sre1. Sre1 regulates the biosynthesis of carotenoids, sterols, and other isoprenoid compounds in *X. dendrorhous*. Despite previous characterizations of several components within the SREBP pathway across various fungal species, little is known about the components of this pathway in *X. dendrorhous*. For example, the precise signal and mechanism triggering Sre1 activation in *X. dendrorhous* are still unknown. In this work, we employed NTG-induced mutagenesis followed by a screening method focused on detecting mutants with altered Sre1 regulation based on pigmentation changes and azole sensitivity. This novel strategy relies on the hypothesis that modifying components of the Sre1 pathway will directly impact carotenoid synthesis and azole resistance, thereby facilitating the identification of mutants and subsequent characterization of the mutated genes in future works. A preliminary round of mutagenesis was performed as a first step to evaluate the effectiveness of the SREBP pathway mutant selection criteria. The 15 potential SREBP mutants selected that exhibited wild-type pigmentation and were sensitive to clotrimazole were further analyzed, including RT-qPCR of a Sre1 target gene (*HMGS*) and sequencing of genes *SRE1* and *STP1*. Importantly, mutations in the *SRE1* and *STP1* genes were detected among the mutants with *HMGS* transcript levels equivalent to those in the wild-type strain. Their phenotype (carotenoid overproduction and resistance to clotrimazole) was restored through complementation assays with Sre1N and either with *SRE1* or *STP1*, depending on each case. As these results validated the screening method for random mutants of the SREBP pathway, in the second round of mutagenesis, only the sequencing of genes *SRE1* and *STP1* was included after mutant selection to avoid mutants harboring mutations in these genes. In this way, 20 mutants (including mutant K20 from the first round of mutagenesis) were selected for further analysis, including whole-genome sequencing and SNP analysis.

From the group of genes affected by high-impact mutations (Figure 4A), Gene Ontology enrichment analysis results included “proteins involved in the import and export of proteins to the nucleus” (P: GO:0006886). Among them, g108 stands out as it encodes a protein from the β-type importin family. In mammals, crystallographic assays have shown that SREBP-2 is imported from the cytoplasm to the nucleus as a dimer, interacting by its bHLH domain with protein importin [56]. Another group of proteins that stand out is “protein-level regulatory proteins”, such as phosphatases and deacetylases. Remarkably, gene g2846 encodes a NAD-dependent histone deacetylase (F: GO:0017136) related to Sir2/SIRT2, which is a member of the sirtuin family with nicotinamide adenine dinucleotide (NAD+)-dependent deacetylase activity. Studies have shown that SIRT2 promotes the SREBP-2 translocation to the nucleus [57], suggesting gene g2846 is a strong candidate to be evaluated for its potential participation in the regulation of Sre1 in *X. dendrorhous*. Similarly, a SAP18 (Sin3 associated polypeptide p18, IPR010516) domain was detected in the polypeptide predicted from g4683, which harbors a high-impact mutation. The Sin3 complex regulates gene expression, is conserved across many eukaryotic organisms, and is associated with histone deacetylases [58]. Thus, a mutation in this gene could affect Sre1 regulation via acetylation/deacetylation, positioning g4683 as a potential candidate involved in Sre1 regulation. Additionally, a group of three kinases encoded by genes g3457 (No GO terms), g1606 (F: GO:0000155), and g5458 (F: GO:0008478), potentially encoding carbohydrate kinase (IPR011611), Histidine kinase/HSP90-like ATPase (IPR003594), and Pyridoxamine kinase/Phosphomethylpyrimidine kinase (IPR013749), respectively, emerged among the genes with high-impact mutations, and g5458 is overexpressed in strain CBS.*FLAG.SRE1N*, which expresses only the active transcription factor Sre1N. Even though, to the best of our knowledge, there are no data linking the role of these specific kinases with the regulation of SREBP in other organisms, a review of the Kinome database mined from UnitProt data indicated that carbohydrate kinase-type proteins, particularly Pyruvate Kinase (PCK1), is a canonical enzyme in the regulation of gluconeogenesis that could phosphorylate protein INSIG [59]. INSIG phosphorylation disrupts the binding of cholesterol-derived oxysterols, activating SREBPs [60]. The group of proteins related to “peptidase function” in the F terms included genes g1695 (F: GO:0008233) and g4290 (F: GO:0004252), which, respectively, encode the type of proteases S8/S53 MEROPS (S8 serine endopeptidase family, subfamilies S8A: subtilisin and S8B: kexin) and S53 (sedolisin), both members of the SB family. These types of peptidases are associated with extracellular degradation, hormonal maturation, and intracellular protein degradation [61], and they could be involved in Sre1 activation or degradation, which is a hypothesis that would be interesting to evaluate in future works. Although the *SRE1* and *STP1* genes were sequenced before the genome sequencing of the analyzed mutants, the gene encoding Stp1 (g3231) also appeared among genes with high-impact mutations, which is responsible for Sre1 cleavage activation in *X. dendrorhous* [11]. Probably we made an involuntary mistake and should not have sequenced the strain harboring this mutation. Another group of genes harboring high-impact mutations includes potential transcription factors that may regulate Sre1 expression. For example, FoxO1, a forkhead box class O transcription factor, regulates the expression of lipogenic genes, including *srebp1*, in mice [62]. SREBP can regulate its expression in several organisms, including *X. dendrorhous* [10]. In this context, a conserved fungal transcription factor domain (IPR007219) and a zinc finger C2H2 superfamily domain (IPR036236) were detected in g4193 and g1730, respectively. These genes exhibit a splicing acceptor (g4193) and a nonsense (g1730) mutation, which may affect their functionality.

Regarding the Gene Ontology enrichment analysis of the 68 genes harboring deleterious moderate-impact mutations, no group related to a molecular function was highlighted for their potential association with SREBP. However, certain genes may indirectly relate to the SREBP pathway (Figure 4B). Firstly, gene g3050 encodes a potential 1-acylglycerol-3-phosphate O-acyltransferase (AGPAT) (F: GO:0003841), which converts glycerol-3-phosphate into 1-acyl-sn-glycerol-3-phosphate. In mice, the AGPAT gene is regulated by SREBP-1 and has three SRE sites at its promoter region [63]. A slight decrease in the transcript levels of SREBP-1c (0.62-fold change) was observed in hippocampal neurons from AGPAT -/- null mice [64], so it is possible that AGPAT might be involved in some way in the transcriptional activation of SREBP. Secondly, gene g3705 encodes a putative dynamin protein, dnm1, a GTPase involved in endocytosis that regulates late events in clathrin-coated vesicle formation. It was reported that while the inhibition of clathrin-dependent endocytosis did not affect the intracellular distribution of cholesterol or the regulation of sterol-sensitive genes, the inhibition of dynamin significantly impacted the regulation of the SREBP-2 gene, among others. Specifically, the inhibition of dynamin led to an accumulation of cholesterol in the late endosomal/lysosomal compartment, affecting its delivery to the ER, where SREBP-2 is regulated by sterol levels highlighting the critical role of dynamin function in cholesterol homeostasis through its effect on SREBP-2 regulation [65]. Thirdly, gene g1377 encodes the 3-hydroxy-3-methylglutaryl-CoA reductase (HMGR) of the mevalonate pathway, and this gene is overexpressed in strain CBS.*FLAG.SRE1N*. Like the protein Scap, HMGR has a sterol sensing domain (SSD), which in HMGR is involved in the sterol-induced degradation of HMGR. The Val32Met missense mutation identified in g1377 lies close to the SSD (IPR000731 85-265), which may affect its sterol-binding affinity. Consequently, this alteration may affect HMGR regulation by sterol levels, contributing to the observed phenotype in the corresponding mutant strain. Although it has been described in other organisms that HMGR degradation and the inhibition of SREBP cleavage may not necessarily involve the same specific sterols [66], there could be a connection between these two processes in *X. dendrorhous*, which could be evaluated in future experiments. Similar to the group of genes with high-impact mutations, among the genes with deleterious moderate-impact mutations, three genes encode potential transcription factors: g1317 (Zinc finger C2H2-type, IPR013087), g5939 (Zinc finger, RING-CH-type, IPR011016), and g3299 (Zinc finger C2H2 superfamily, IPR036236), which could be related to the regulation of Sre1 at its expression level or interact with Sre1 for its function in *X. dendrorhous*. Another candidate gene is g5928, which encodes the yeast cytochrome P450 oxidoreductase (CPR) protein and is overexpressed in strain CBS.*FLAG.SRE1N*. Cytochrome P450 oxidoreductase is required for the synthesis of astaxanthin and ergosterol in *X. dendrorhous*, as both pathways include cytochrome P450 proteins that require a redox partner for their activity, a role fulfilled by CPR [67,68,69]. For this reason, a mutation in CPR might impact ergosterol biosynthesis, and therefore, a mutation in g5928 might affect Sre1 activation in *X. dendrorhous*. Genes potentially encoding proteins involved in the transport of macromolecules between the cytoplasm and nucleus were also identified among genes with deleterious moderate-impact mutations. These include g6171 (Importin-beta, N-terminal domain, IPR001494) and g5199 (Importin-beta, N-terminal domain, IPR001494), which could be related to the import or export of Sre1 to or from the nucleus. In addition, a coatomer subunit gamma C-terminal domain (IPR032154) was identified in the potential polypeptide encoded by g2728, which could be involved in retrograde transport from the Golgi apparatus to the ER, where COPI (coat protein complex I) vesicles are the primary carriers. In *A. nidulans*, SrbA proteolytic activation by SppA, which localizes to the ER, requires prior Dsc complex-dependent proteolysis [21]. However, the localization of the Dsc complex was not possible to determine [21]. In *S. pombe*, the Dsc complex resides in the Golgi apparatus and is also required for Sre1 activation [43]. Therefore, it is possible that SrbA could be transported to the Golgi apparatus for Dsc complex-dependent proteolysis and then transported back to the ER for SppA cleavage, for which the COPI-trafficking system could be required [21]. A similar mechanism involving complex cellular trafficking systems could operate in *X. dendrorhous* for Sre1 activation. Finally, three genes potentially related to the ubiquitin-proteasome system had deleterious moderate-mutations: g3358 (Csn12 family, IPR045114), g6320 (Proteasome, subunit alpha/beta, IPR001353), and g6004 (E3 ubiquitin ligase, domain of unknown function DUF908, IPR010309). The ubiquitin–proteasome system is involved in the turnover of SREBPs, where polyubiquitination of SREBPs leads to their proteasome-dependent degradation [70]. Considering that the phenotype selected in the mutants studied in our work aligns with an absence of activity of the active form of Sre1, mutants of the Sre1 degradation pathway would not be expected. However, it is also possible that mutations in its degradation pathway could promote the degradation of the active transcription factor. This situation should be studied further. Furthermore, two strains had mutations in the gene g904 (F: GO:0003838): one had a high-impact mutation, and the other a deleterious moderate-impact mutation. This gene encodes the sterol 24-C-methyltransferase (Erg6) involved in ergosterol biosynthesis. Therefore, mutations in this gene may result in a strain unable to produce ergosterol, potentially leading to the accumulation of zymosterol. In *X. dendrorhous*, previous work suggests that it is not the absence of ergosterol that triggers the activation of Sre1 but rather the accumulation of sterols other than ergosterol in the CBS.*cyp61^-^* mutant [71]. These sterols may not be produced in g904 mutants, potentially resulting in defective Sre1 activation.

**Figure 5:**
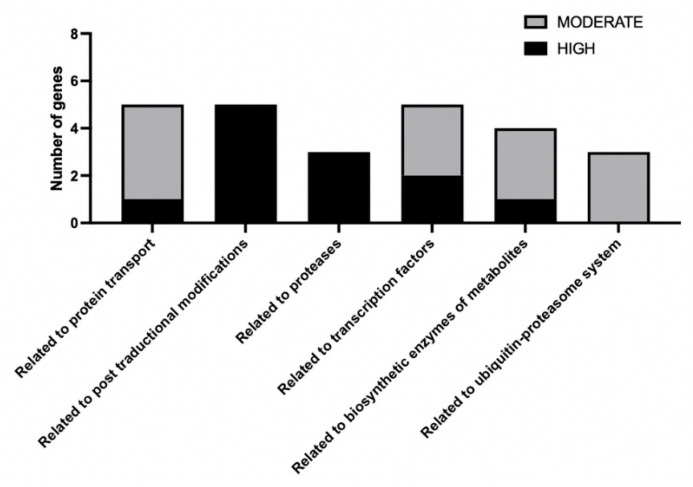
Classification of the 25 main candidate genes. The 25 genes mentioned as candidates for participating in the regulation of Sre1 in *X. dendrorhous* were classified based on their functions into the categories related to protein trafficking and transport, post-translational modification, proteases, transcription factors, biosynthetic enzymes of metabolites, and the ubiquitin–proteasome system. The number of genes with high-impact mutations is represented in black, and those with deleterious moderate-impact mutations in gray.

Finally, as a summary, based on all the genes mentioned in the discussion, these were grouped according to their predicted functions related to (i) protein trafficking and transport (one gene with HIGH- and four with deleterious moderate-impact mutations), (ii) post-translational modification (five genes with high-impact mutations), (iii) proteases (three genes with high-impact mutations), (iv) transcription factors (two genes with high- and three genes with deleterious moderate-impact mutations), (v) biosynthetic enzymes of metabolites (one gene with high- and three genes with deleterious moderate-impact mutations), and (vi) the ubiquitin–proteasome system (three genes with deleterious moderate-impact mutations) (Figure 5).

## 5. Conclusions

A screening strategy to select mutants associated with the SREBP pathway in *X. dendrorhous* was developed using two main criteria: pigmentation changes and clotrimazole sensitivity. This strategy led to the selection of 20 mutants. Subsequent sequencing and SNP analysis unveiled 116 candidate genes, comprising 49 genes with high-impact mutations and 68 with deleterious moderate-impact mutations. Bioinformatic analyses, including BLAST, InterProScan, and Gene Ontology enrichment analysis, allowed us to propose 25 genes as potential participants in regulating the SREBP pathway in *X. dendrorhous*. The key findings of this study include identifying genes potentially encoding proteins implicated in protein import/export to the nucleus, sterol biosynthesis, protein regulatory activities such as deacetylases, the ubiquitin–proteasome system, and a subset of kinases potentially influencing SREBP regulation. Additionally, certain peptidases were hypothesized to be associated with Sre1 activation and transcription factors that could be involved in *SRE1* regulation or act together with Sre1N. This study establishes valuable groundwork for further exploration into the molecular mechanisms controlling the regulation of the SREBP pathway in *X. dendrorhous*.

## Figures and Tables

**Table 1:** Strains and plasmids used in this work.

Strain/Plasmid	Description	Reference
Strains		
DH5α	Strain of *E. coli* used for molecular cloning and plasmid propagation.	[32]
CBS 6938	*X. dendrorhous* wild-type strain. Strain with wild-type pigmentation and resistant to clotrimazole.	ATCC 96594
CBS.*cyp61^-^*	Mutant derived from CBS 6938. The *CYP61* locus was interrupted by the zeocin resistance cassette. This strain overproduces carotenoids and is resistant to clotrimazole.	[12]
CBS.*cyp61^-^.FLAG.SRE1*	Mutant derived from CBS 6938. The native *SRE1* gene was replaced by a gene variant that expresses the Sre1 protein fused to the 3xFLAG epitope at its N-terminus, followed by the zeocin resistance cassette used for transformant selection. This strain overproduces carotenoids and is resistant to clotrimazole.	[9]
G4, K8, C3, Ñ1, A14, A2, Ñ34, K21, T1, X4, Ñ18, T16, K20, M6 and D1	Initial 15 selected strains obtained by NTG mutagenesis from CBS.*cyp61^-^* with a wild-type pigmentation and sensible to clotrimazole. Strain K20 was included for whole-genome sequencing and SNP analysis (Sample_K20).	This work
001-019	Nineteen strains obtained by NTG mutagenesis from CBS.*cyp61^-^.FLAG.SRE1*, with a wild-type pigmentation and sensible to clotrimazole. These strains were used for whole-genome sequencing and SNP analysis (Samples 001-019).	This work
Plasmids		
pBluescript SK- (pBS)	Cloning vector	Agilent Technologies Inc.
pBS-*gSTP1*^up-down^	Plasmid used to replace the *X. dendrorhous STP1* gene with the wild-type sequence of the gene by homologous recombination in strains obtained by random mutagenesis.	[11]
pXd-*gSRE1*-zeo	Plasmid used to replace the *X. dendrorhous SRE1* gene with the wild-type sequence of the gene by homologous recombination in strains obtained by random mutagenesis.	[11]
pXd-*gSRE1N*-zeo	Plasmid used to replace the *X. dendrorhous SRE1* gene with the gene variant that expresses Sre1N (Sre1 N-terminal domain) by homologous recombination in strains obtained by random mutagenesis.	[9]

**Table 2:** Summary of the results from the analyses of mutants derived from strain CBS.*cyp61^-^*.

Mutant	*HMGS* Transcript Levels Compared to wt Strain	*Sre1N* Complementation	Mutation in Genes (Sequencing)	*SRE1* Complementation	*STP1* Complementation
G4	similar	Yes	*SRE1*	Yes	-
K8	similar	No	-	-	-
C3	upregulated	-	-	-	-
Ñ1	similar	Yes	*STP1*	No	Yes
A14	upregulated	-	-	-	-
A2	upregulated	-	-	-	-
**Ñ34**	similar	Yes	None	No	No
K21	similar	Yes	*STP1*	No	Yes
T1	similar	Yes	*STP1*	No	Yes
X4	downregulated	-	-	-	-
Ñ18	downregulated	-	*-*	-	-
T16	downregulated	-	-	-	-
**K20**	Similar	Yes	None	No	No
M6	downregulated	-	-	-	-
D1	similar	Yes	*STP1*	No	Yes

Mutants with a wild-type pigmentation and sensible to clotrimazole. “wt”: wild-type; “Yes” or “No” indicates if the introduction of the corresponding gene reversed or did not reverse the phenotype to the one observed in strain CBS.*cyp61^-^* in terms of pigmentation and resistance to clotrimazole; (-): analysis was not conducted as the mutant was discarded based on the previous selection criteria.

**Table 3:** Characterization of the domains and RNA-seq analysis of the SNPs identified as high-impact.

Gene Code (#)	Protein Effect	Blast Hit (Protein Ref_seq)	InterProScan Hit	Log2 Change	*p*-Value Adjust
g904	Arg288 *	delta-sterol C-methyltransferase	Erg6/SMT methyltransferase (IPR050447)	**4.48 × 10^0^**	**4.28 × 10^−36^**
g5458	Trp371 *	pyridoxal kinase	Pyridoxamine kinase/Phosphomethylpyrimidine kinase (IPR013749)	**1.47 × 10^0^**	**2.75× 10^−9^**
g5041	Splicing	ND	ND	**1.29 × 10^0^**	**1.11 × 10^−7^**
g751	Splicing	ND	ND	**1.05 × 10^0^**	**1.05 × 10^−5^**
g2375	Splicing	ND	ND	**1.02 × 10^0^**	**6.45 × 10^−3^**
g6106	Glu46 *	ND	ND	8.70 × 10^−1^	4.92 × 10^−1^
g5290	Arg480 *	ND	Chromatin SPT2 (IPR013256)	7.75 × 10^−1^	5.10 × 10^−3^
g1695	Met1?	predicted protein	Signal peptidase complex subunit 2 (IPR009582)	7.09 × 10^−1^	2.79 × 10^−3^
g6091	Splicing	urea transporter	Sodium/solute symporter (IPR001734)	6.84 × 10^−1^	1.10 × 10^−1^
g3457	Splicing	Ribokinase-like protein	Carbohydrate kinase PfkB (IPR011611)	6.64 × 10^−1^	1.28 × 10^−2^
g2927	Leu248 *	ND	ND	6.10 × 10^−1^	1.72 × 10^−1^
g5215	Splicing	glutathione peroxidase	Glutathione peroxidase (IPR000889)	5.52 × 10^−1^	9.42 × 10^−3^
g4522	Met1?	hypothetical protein TREMEDRAFT_69977	Protein Zds1, C-terminal (IPR013941)	3.78 × 10^−1^	1.65 × 10^−1^
g3946	Splicing	2-acylglycerol O-acyltransferase 2	Diacylglycerol acyl transferase (IPR007130)	3.38 × 10^−1^	2.36 × 10^−1^
g4324 (2)	Splicing (both)	predicted protein	ND	2.67 × 10^−1^	1.88 × 10^−1^
g3664	Trp16 *	hypothetical protein TREMEDRAFT_73274	ND	2.58 × 10^−1^	4.42 × 10^−1^
g108	Splicing	ARM repeat-containing protein	Exportin-1/Importin-beta-like (IPR013598)	1.66 × 10^−1^	4.75 × 10^−1^
g671	Splicing	electron-transferring-flavoprotein dehydrogenase	ND	1.58 × 10^−1^	6.36 × 10^−1^
g716	Splicing	Mov34-domain-containing protein	Cop9 signalosome subunit 5C-terminal domain (IPR040961)	1.32 × 10^−1^	6.27 × 10^−1^
g3597	Splicing	predicted protein	ND	7.96 × 10^−2^	8.89 × 10^−1^
g2198	Splicing	DNA repair protein	ERCC4 domain (IPR006166)	6.56 × 10^−2^	8.35 × 10^−1^
g5577 (2)	Gln669 * Trp724 *	transcription factor	Ankyrin repeat-containing domain superfamily (IPR036770)	4.79 × 10^−3^	9.90 × 10^−1^
g3833	Splicing	ND	ND	−5.05 × 10^−2^	8.92 × 10^−1^
g5637	Splicing	membrane organization and biogenesis-related protein	TB2/DP1/HVA22-related protein (IPR004345)	−7.98 × 10^−2^	7.78 × 10^−1^
g4193	Splicing	hypothetical protein I206_05628	Transcription factor domain, fungi (IPR007219)	−9.16 × 10^−2^	8.37 × 10^−1^
g3231	Trp239 *	hypothetical protein TRAVEDRAFT_171989	Peptidase M50 (IPR008915)	−9.57 × 10^−2^	7.89 × 10^−1^
g2846	Splicing	NAD-dependent protein deacetylase sirtuin-2-like	Sirtuin, class II (IPR026587)	−2.05 × 10^−1^	3.30 × 10^−1^
g4683	Met1?	predicted protein	Sin3 associated polypeptide p18 (IPR010516)	−2.68 × 10^−1^	4.21 × 10^−1^
g3594	Trp641 *	vacuolar membrane protein, putative	PQ-loop repeat (IPR006603)	−2.79 × 10^−1^	1.35 × 10^−1^
g2091	Splicing	acid phosphatase, putative	Histidine phosphatase superfamily, clade-2 (IPR000560)	−3.96 × 10^−1^	2.53 × 10^−1^
g6351	Trp489 *	ND	Pentatrico peptide repeat (IPR002885)	−4.42 × 10^−1^	8.19 × 10^−2^
g1730	Trp413 *	hypothetical protein EHS24_009014	Zinc finger C2H2-type (IPR013087)	−4.94 × 10^−1^	2.25 × 10^−2^
g2575	Trp241 *	kinesin-domain-containing protein	Kinesin motor domain (IPR001752)	−5.01 × 10^−1^	1.17 × 10^−2^
g1263	Splicing	predicted protein	ND	−6.28 × 10^−1^	7.80 × 10^−3^
g1367	Trp254 *	glucocorticoid receptor-like protein	ND	−6.97 × 10^−1^	2.01 × 10^−2^
g3954	Cys548 *	hypothetical protein L202_04737	ND	−7.10 × 10^−1^	7.20 × 10^−5^
g5918	Splicing	predicted protein	Integral membrane bound transporter domain (IPR049453)	−7.59 × 10^−1^	7.59 × 10^−4^
g6306	Arg929 *	RNB-domain-containing protein	Nucleic acid-binding, OB-fold (IPR012340)	−8.12 × 10^−1^	4.54 × 10^−4^
g4290	Splicing	serine-type endopeptidase, putative	Peptidase S8/S53 domain superfamily (IPR036852)	−9.27 × 10^−1^	6.99 × 10^−9^
g4519	Splicing	amino acid transporter, putative	Amino acid permease/SLC12A domain (IPR004841)	−9.82 × 10^−1^	1.55 × 10^−4^
g1606	Lys1419 *	multi-sensor hybrid histidine kinase	Histidine kinase/HSP90-like ATPase (IPR003594)	−1.01 × 10^0^	6.96 × 10^−5^
g5825	Trp373 *	glucan endo-1,3-alpha-glucosidase agn1	Glycoside hydrolase family 71 (IPR005197)	−1.25 × 10^0^	2.04 × 10^−7^
g912	Splicing	putative alcohol dehydrogenase	Alcohol dehydrogenase-like, N-terminal (IPR013154)	−1.28 × 10^0^	1.21 × 10^−9^
g5575	Splicing	signal sequence binding protein	Sortilin, C-terminal (IPR031777)	−1.55 × 10^0^	1.04 × 10^−14^
g890	Splicing	putative alcohol dehydrogenase	Alcohol dehydrogenase-like, C-terminal (IPR013149)	−1.82 × 10^0^	1.42 × 10^−15^
g693	Splicing	predicted protein	Band7/SPFH domain superfamily (IPR036013)	−2.20 × 10^0^	2.31 × 10^−15^
g1908	Trp59 *	glutamine synthetase	Glutamine synthetase, N-terminal domain superfamily (IPR036651)	−2.63 × 10^0^	9.99 × 10^−25^
g5769	Glu626 *	DDE-type integrase/transposase/recombinase	Zinc finger, CCHC-type (IPR001878)	−3.16 × 10^0^	1.21 × 10^−22^
g4483	Gln126 *	dihydrodipicolinate synthase	ND	−3.59 × 10^0^	1.41 × 10^−24^

“(#)”: number of different high-impact mutations in the corresponding gene; “*”: stop codon; “Met1?”: substitution affecting the translation initiation codon (Met1). The consequence of this change is not predictable. “ND”: not detected in the analysis. Genes were ordered based on the Log2 change between strain CBS.*FLAG.SRE1N* vs. the wild-type, from highest to lowest, with genes upregulated by Sre1 highlighted in bold.

**Table 4:** Characterization of the domains and RNA-seq analysis of the SNPs identified as moderate-impact.

Gene Code (#)	Protein Effect	Blast Hit	InterProScan Hit	Log2 Change	*p*-Value Adjust
g904	Pro265Leu	delta-sterol C-methyltransferase	Erg6/SMT methyltransferase (IPR050447)	**4.48 × 10^0^**	**4.28 × 10^−36^**
g5928	Val363Met	cytochrome P450 oxidoreductase	NADPH-cytochrome P450 reductase (IPR023208)	**2.56 × 10^0^**	**1.19 × 10^−13^**
g6107 (5)	Gly263Arg Ala209Val Ala203Asp Gly171Glu Gly3Glu	ATP synthase F0 subunit 6 (mitochondrion)	ATP synthase, F0 complex, subunit A (IPR000568)	**2.56 × 10^0^**	**1.49 × 10^−1^**
g1377	Val32Met	hydroxymethylglutaryl-CoA reductase (NADPH)	Hydroxymethylglutaryl-CoA reductase, class I/II (IPR002202)	**1.49 × 10^0^**	**4.40 × 10^−9^**
g5142	Val805Ile	ribosome biogenesis protein tsr1	Ribosome biogenesis protein BMS1/TSR1, C-terminal (IPR007034)	**1.40 × 10^0^**	**2.07 × 10^−6^**
g1155	Ala310Val	P-loop containing nucleoside triphosphate hydrolase protein	P-loop containing nucleoside triphosphate hydrolase (IPR027417)	**1.32 × 10^0^**	**6.28× 10^−9^**
g3604	Pro158Leu	ribulose-5-phosphate 3-epimerase	Ribulose-phosphate 3-epimerase-like (IPR000056)	**1.31 × 10^0^**	**8.59× 10^−7^**
g5830	Thr68Ile	FK506-binding protein 2	N/D	**1.23 × 10^0^**	**1.02 × 10^−4^**
g4594	Gly246Asp	pyruvate kinase	Pyruvate kinase, C-terminal (IPR015795)	**1.08 × 10^0^**	**9.34 × 10^−3^**
g2552	Val68Ile	Phosphoribosyl aminoimidazole carboxamide formyl transferase/IMP cyclohydrolase	Bifunctional purine biosynthesis protein PurH-like (IPR002695)	**1.08 × 10^0^**	**2.17 × 10^−4^**
g368	Ser6Ile	1-alkyl-2-acetyl glycerol phospho choline esterase	WD40 repeat (IPR001680)	**1.01 × 10^0^**	**5.35 × 10^−6^**
g3129	Tyr686Cys	ATP-dependent RNA helicase DDX35	Helicase associated domain (HA2), winged-helix domain (IPR048333)	9.80 × 10^−1^	8.76 × 10^−5^
g2716	Pro39Leu	U3 sno RNP-associated protein Rrp5	Tetratricopeptide-like helical domain superfamily (IPR011990)	9.80 × 10^−1^	1.65 × 10^−4^
g402	Pro596Ser	dimethylaniline monooxygenase (N-oxide forming)	FAD/NAD(P)-binding domain superfamily (IPR036188)	6.10 × 10^−1^	1.76 × 10^−2^
g378	Val58Met	ribose-phosphate pyrophosphokinase	Phosphoribosyl transferase-like (IPR029057)	5.37 × 10^−1^	1.64 × 10^−2^
g3461	Arg132Gln	putative CDC12-septin	P-loop containing nucleoside triphosphate hydrolase (IPR027417)	4.73 × 10^−1^	4.02 × 10^−2^
g6171	Val532Met	importin-alpha export receptor	Importin-beta, N-terminal domain (IPR001494)	4.61 × 10^−1^	5.93 × 10^−2^
g2350	Pro158Leu	leukotriene A-4 hydrolase/aminopeptidase	Aminopeptidase N-like, N-terminal domain (IPR045357)	4.41 × 10^−1^	2.23 × 10^−2^
g3216	Val3010Ile	Midasin	Von Willebrand factor A-like domain superfamily (IPR036465)	4.38 × 10^−1^	2.03 × 10^−1^
g5516	Ile204Met	gamma-tubulin complex component 3	Gamma tubulin complex component protein, N-terminal (IPR041470)	3.83 × 10^−1^	1.04 × 10^−1^
g4453	Ser987Phe	ribosome assembly protein 1	Translation protein, beta-barrel domain superfamily (IPR009000)	2.99 × 10^−1^	1.51 × 10^−1^
g5781	Pro70Ser	DEAD-domain-containing protein	Helicase, C-terminal domain-like (IPR001650)	2.85 × 10^−1^	2.02 × 10^−1^
g3358	Thr358Ile	predicted protein	Csn12 family (IPR045114)	2.24 × 10^−1^	4.31 × 10^−1^
g6320	Pro14Ser	proteasome subunit alpha type 4, putative	Proteasome, subunit alpha/beta (IPR001353)	1.95 × 10^−1^	3.95 × 10^−1^
g1143	Thr63Ile	allantoicase, putative	Rml C-like cupin domain superfamily (IPR011051)	1.78 × 10^−1^	5.05 × 10^−1^
g1906	Asn146Ser	L-fucose transporter	MFS transporter superfamily (IPR036259)	1.78 × 10^−1^	5.57 × 10^−1^
g1317	Pro435Ser	hypothetical protein GLOIN_2v1436072, partial	Zinc finger C2H2-type (IPR013087)	9.64 × 10^−2^	7.96 × 10^−1^
g4775	Met82Ile	pyruvate dehydrogenase (acetyl-transferring) E1 component, alpha subunit	Pyruvate dehydrogenase (acetyl-transferring) E1 component, alpha subunit, subgroup y (IPR017597)	5.48 × 10^−2^	9.00 × 10^−1^
g2238	Val1270Ile	PAB-dependent poly(A)-specific ribonuclease subunit PAN2	WD40-repeat-containing domain superfamily (IPR036322)	−3.84 × 10^−2^	8.75 × 10^−1^
g5211	Val213Phe	protein arginine N-methyltransferase	S-adenosyl-L-methionine-dependent methyltransferase superfamily (IPR029063)	−8.02 × 10^−2^	8.85 × 10^−1^
g2877	Thr322Ile	DNA polymerase zeta subunit	Ribonuclease H-like superfamily (IPR012337)	−9.34 × 10^−2^	7.57 × 10^−1^
g5939	Leu1427Phe	hypothetical protein PUNSTDRAFT_35157, partial	Zinc finger, RING-CH-type (IPR011016)	−1.00 × 10^−1^	7.56 × 10^−1^
g2572	Gly125Ser	biotin-[acetyl-CoA-carboxylase] ligase	Class II Aminoacyl-tRNA synthetase/Biotinyl protein ligase (BPL) and lipoyl protein ligase (LPL) (IPR045864)	−1.37 × 10^−1^	6.32 × 10^−1^
g4518	Ala331Thr	PLP-dependent transferase	Pyridoxal phosphate-dependent transferase (IPR015424)	−1.67 × 10^−1^	5.77 × 10^−1^
g2586	Val445Ile	ATPase, V1 complex, subunit H	Armadillo-type fold (IPR016024)	−1.70 × 10^−1^	6.08 × 10^−1^
g5199	Trp665Arg	CRM1 C terminal-domain-containing protein	Importin-beta, N-terminal domain (IPR001494)	−1.71 × 10^−1^	5.88 × 10^−1^
g3195	Val106Met	replication factor C subunit 3/5	DNA polymerase III, clamp loader complex, gamma/delta/delta subunit, C-terminal (IPR008921)	−1.84 × 10^−1^	5.81 × 10^−1^
g4523	Gly63Asp	Golgi phosphoprotein 3	Golgi phosphoprotein 3-like (IPR008628)	−2.92 × 10^−1^	3.03 × 10^−1^
g3705	Pro154Ser	dynamin protein dnm1, putative	Dynamin, N-terminal (IPR045063)	−3.03 × 10^−1^	2.22 × 10^−1^
g3050 (2)	Ile10Ser Asn11Asp	1-acylglycerol-3-phosphate O-acyltransferase, putative	1-acyl-sn-glycerol-3-phosphate acyl transferase (IPR004552)	−3.04 × 10^−1^	4.53 × 10^−1^
g3734	Ser747Phe	kinesin-domain-containing protein	Kinesin motor domain (IPR001752)	−3.30 × 10^−1^	5.77 × 10^−1^
g1433	Ser91Phe	chaperone, putative	Tetratricopeptide repeat (IPR019734)	−3.80 × 10^−1^	1.72 × 10^−1^
g2728	Asp797Asn	adaptin N terminal region-domain-containing protein	Coatomer subunit gamma, C-terminal (IPR032154)	−3.93 × 10^−1^	2.40 × 10^−1^
g3299	Val439Met	S-adenosyl-L-methionine-dependent methyltransferase	Zinc finger C2H2 superfamily (IPR036236)	−4.07 × 10^−1^	1.10 × 10^−1^
g5836	Gly74Glu	peptidyl-prolyl cis-trans isomerase B	Cyclophilin-like domain superfamily (IPR029000)	−4.53 × 10^−1^	5.95 × 10^−2^
g4740	Gly42Arg	ATPase GET3	Anion-transporting ATPase-like domain (IPR025723)	−4.75 × 10^−1^	2.06 × 10^−1^
g4621	Ala48Thr	homocitrate synthase	Pyruvate carboxyltransferase (IPR000891)	−4.97 × 10^−1^	3.62 × 10^−2^
g2740	Ala62Val	hypothetical protein SCHCODRAFT_79669	Aldehyde dehydrogenase domain (IPR015590)	−5.00 × 10^−1^	1.71 × 10^−2^
g3165	Asp98Asn	Enolase	Enolase (IPR000941)	−5.13 × 10^−1^	5.18 × 10^−2^
g3166	Thr388Ile	dihydroxy-acid dehydratase	Dihydroxy-acid dehydratase (IPR004404)	−5.18 × 10^−1^	9.54 × 10^−2^
g3697	Arg295Gln	SPOC domain-like protein	Ku, C-terminal (IPR014893)	−5.27 × 10^−1^	2.18 × 10^−2^
g1933	Thr278Ile	ATP-dependent RNA helicase eIF4A	Helicase, C-terminal domain-like (IPR001650)	−6.23 × 10^−1^	4.05 × 10^−2^
g6193	Ala163Thr	predicted protein	Armadillo-type fold (IPR016024)	−7.16 × 10^−1^	7.89 × 10^−2^
g630	Ala71Thr	peroxisomal assembly protein PEX3	Peroxin-3 (IPR006966)	−8.89 × 10^−1^	8.92 × 10^−5^
g4137	Asp574Asn	vacuolar protein sorting protein (VPS11), putative	Vacuolar protein sorting protein 11 C-terminal (IPR024763)	−9.29 × 10^−1^	1.33 × 10^−3^
g4083	Val245Ile	GTP-binding protein TypA	EF-G domain III/V-like (IPR035647)	−9.93 × 10^−1^	2.31 × 10^−3^
g767	Glu472Lys	glycogen(starch) synthase	N/D	−1.05 × 10^0^	5.17 × 10^−3^
g3532	Thr252Ile	Arginase	Ureohydrolase domain superfamily (IPR023696)	−1.10 × 10^0^	7.19 × 10^−5^
g2334	Val266Met	acetyl-CoA acyltransferase	Thiolase, N-terminal (IPR020616)	−1.14 × 10^0^	5.15 × 10^−4^
g3912	Thr184Pro	cyclin-dependent protein kinase inhibitor	SPX domain (IPR004331)	−1.31 × 10^0^	7.10 × 10^−5^
g3916	Gly112Arg	DNA repair protein RAD51	DNA recombination and repair protein Rad51-like, C-terminal (IPR013632)	−1.50 × 10^0^	7.27 × 10^−4^
g927	Gly506Glu	heat shock protein 70	ATPase, nucleotide binding domain (IPR043129)	−1.51 × 10^0^	2.25 × 10^−3^
g304	Val201Phe	hexose transport-related protein, putative	Major facilitator, sugar transporter-like (IPR005828)	−1.52 × 10^0^	1.27 × 10^−5^
g4590	Ser36Tyr	putative 60S ribosomal protein L19	Large ribosomal subunit protein L19 domain (IPR000196)	−1.53 × 10^0^	3.05 × 10^−7^
g683	Gly5Asp	solute carrier family 36 (proton-coupled amino acid transporter)	Metalloenzyme, LuxS/M16 peptidase-like (IPR011249)	−1.69 × 10^0^	4.15 × 10^−6^
g6004 (2)	Leu906Phe Val3038Met	E3 ubiquitin-protein ligase ptr1	E3 ubiquitin ligase, domain of unknown function DUF908 (IPR010309)	−1.98 × 10^0^	7.90 × 10^−9^
g1436	Ser64Leu	Glucan endo-1,3-alpha-glucosidase agn1	Carbohydrate-binding WSC (IPR002889)	−2.57 × 10^0^	5.10 × 10^−16^
g5769	Gln277Pro	DDE-type integrase/transposase/recombinase	Zinc finger, CCHC-type (IPR001878)	−3.16 × 10^0^	1.21 × 10^−22^

“(#)”: number of different deleterious moderate-impact mutations in the corresponding gene; “ND”: not detected in the analysis. Genes are ordered based on the Log2 change between strain CBS.*FLAG.SRE1N* vs. the wild-type, from highest to lowest, with genes upregulated by Sre1 highlighted in bold.

## Data Availability

Reference Genome Shotgun project strain CBS 6938 (accession number: GCA_001007165.2). Reads of mutants 001-019 and K20 (accession number: PRJNA1098865). RNA-seq data (accession number: GSE152739).

## Supplementary Materials

The following supporting information can be downloaded at: https://www.mdpi.com/article/10.3390/biom14070778/s1, Figure S1. Gene Ontology analysis of the 49 genes with high-impact mutations (cellular component terms, C: terms). Figure S2. Gene Ontology analysis of the 49 genes with high-impact mutations (molecular function terms, F: terms). Figure S3. Gene Ontology analysis of the 49 genes with high-impact mutations (biological process terms, P: terms). Figure S4. Gene Ontology analysis of the 68 genes with moderate-impact mutations (cellular component terms, C: terms). Figure S5. Gene Ontology analysis of the 68 genes with moderate-impact mutations (molecular function terms, F: terms). Figure S6. Gene Ontology analysis of the 68 genes with moderate-impact mutations (biological process terms, P: terms). Table S1. Primers used in this work. Table S2. Classification of mutations by impact levels with SnpEff. Table S3. Total SNVs identified in this work. Table S4. Total SNPs after the filters mentioned in this work. Table S5. Total high-impact SNPs and functional annotations. Table S6. Total moderate-impact SNPs and functional annotations. Table S7. Statistical analysis with DESeq2 for genes with high- and deleterious moderate-impact mutations. Table S8. Annotation of the genome GCA_014706385.1 using BRAKER and BLAST (Protein Refseq).
